# Effect of Salicylic Acid Pre-Treatment after Long-Term Desiccation in the Moss *Syntrichia ruralis* (Hedw.) Web. and Mohr

**DOI:** 10.3390/plants9091097

**Published:** 2020-08-26

**Authors:** Ruchika, Zsolt Csintalan, Evelin Ramóna Péli

**Affiliations:** 1 Doctoral School of Biological Sciences, Institute of Botany and Ecophysiology, Szent István University, Páter Károly utca 1., H-2100 Gödöllő, Hungary; csintalan.zsolt@mkk.szie.hu; 2 Department of Botany, University of Veterinary Medicine, István utca. 2., H-1078 Budapest, Hungary

**Keywords:** chlorophyll fluorescence, antioxidant enzymes, desiccation, photosystem, salicylic acid

## Abstract

The main objective of this research was to examine the effects of exogenous salicylic acid (SA) and to study the seasonal variation of the chlorophyll *a* fluorescence parameters and antioxidant enzymatic activities in desiccation-tolerant moss species *Syntrichia ruralis* (Hedw.) Web. and Mohr. Aqueous 0.001 M SA solution was sprayed on the moss cushions collected from semi-arid sandy grassland, Hungary in three seasons (spring, summer, autumn). These cushions were kept under the observation for 10 Days. Chlorophyll *a* fluorescence parameters, i.e., maximum photochemical quantum yield of PS II (Fv/Fm), effective photochemical quantum yield of PS II (ΦPSII), non-photochemical quenching (NPQ), and antioxidant enzymatic activities, i.e., ascorbate peroxidase (APX), catalase (CAT), guaiacol peroxidase (POD), and protein content were determined. The results showed the increase of Fv/Fm in spring and autumn season while ΦPSII was reduced significantly during spring and summer season after treatment with SA compared to control. SA-treated mosses showed higher values of non-photochemical quenching (NPQ) during the spring and autumn season than in summer. Activities of enzyme APX and CAT were found to increase in SA-treated except POD activity. In SA-treated moss cushions, lower protein content was found. It can be concluded that seasonal variation has been observed in chlorophyll fluorescence and antioxidant system after long term of desiccation in *S. ruralis* species that could be because of SA and might be due to fluctuations in conditions of their habitat, duration of light intensity, temperature and precipitation.

## 1. Introduction

Bryophytes are the experimental model organisms that contribute to study of evolution of plant hormones. Phytohormones are low-molecular-weight molecules that activate an effective defense response against both biotic and abiotic environmental stresses and responsible for growth and development. In bryophytes, only auxins, cytokinins, and abscisic acid has been earlier investigated. However, there is limited information related to the occurrence, metabolism, and function of plant hormones in mosses [1]. Most of the previous investigations concerning the effect of phytohormones on bryophyte were focused on the model moss *Physcomitrella patens* (Hedw.) Bruch and Schimp., and *Funaria hygrometrica* (Hedw.), and few reports are conducted on the other bryophytes. Salicylic acid (SA) can inhibit the later stages of bud formation in *F. hygrometrica* in a dose-dependent manner. Results indicated that these mosses might use SA as developmental signals [2]. However, further research is needed to clarify and understand their distribution and to study the mechanism behind signal transduction in bryophytes [3]. In vascular plants, SA is a plant growth regulator that plays an important role in seed germination and development, cell growth, stomatal closure and enhancement of enzyme activity against a variety of biotic and abiotic stresses through various morphological, physiological and biochemical mechanisms [4]. These environmental stresses lead to the production and accumulation of reactive oxygen species (ROS) in various cell organelles [5]. In plants, catalase and peroxidases help in scavenging ROS and provide protection against oxidative damage which causes inactivation of cell functions [6]. Catalase is an oxidoreductase enzyme which decomposes H_2_O_2_ to water and molecular oxygen [7]. Peroxidase activities play protective roles in enhancing their tolerance under unfavorable conditions [8].

Bryophytes are sensitive plants that produce secondary metabolites that strengthen them with strong antioxidative machinery to cope with biotic and abiotic stress [9]. The antioxidant defense response protects the cell organelles and cell membranes against oxidative damage. Overproduction of ROS disrupting the structure of the cell and reacts with lipids and proteins leads to cell damage under unfavorable conditions [10]. Earlier reports on seasonal variations in chlorophyll *a* fluorescence in *S. ruralis* (Hedw.) Web. and Mohr. [11] indicated the variations due to different degrees of environmental stresses such as drought, heat, and variation in extreme temperatures. By measuring the yield of chlorophyll fluorescence, the photochemical efficiency and the degree of heat dissipation could be estimated. The increasing photosynthesis will lead to a decrease in energy dissipation [12].

In this study, we examined the level of plant stress in desiccation tolerant bryophyte; *S. ruralis* (synonym: *Tortula ruralis*) that belongs to the family of Pottiaceae. It is also known as sandhill screw moss that is found abundantly in the form of extensive mats in open exposed areas of sandy dunes in a semi-arid grassland [13]. We carried out the measurements of the chlorophyll *a* fluorescence parameter and examined its seasonal variation to study the functioning of photosynthetic apparatus and associated photoprotective mechanism in bryophytes caused due to SA pre-treatment. Here, we investigated the impacts of exogenous SA pre-treatment on the activities of antioxidant enzymes: Ascorbate peroxidase (APX), catalase (CAT), and guaiacol peroxidase (POD) in *S. ruralis* during the different three seasons (spring, summer, autumn).

## 2. Results

### 2.1. Determination of Water Content (WC%) under SA Treatment during Rehydration Period

**Figure 1:**
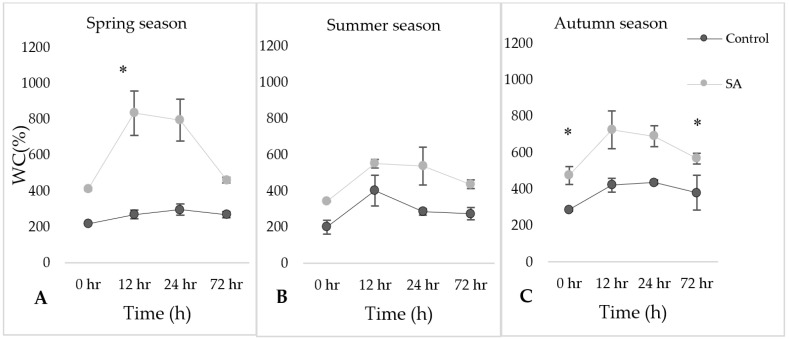
The water content percentage (WC%) in the leaves of *Syntrichia ruralis* after salicylic acid (SA) treatment during rehydration period under SA treatment in different seasons ((**A**)—Spring, (**B**)—Summer, (**C**)—Autumn). The mean values (*n* = 3) ± SD marked with an asterisk (*) are significantly different at *p* ≤ 0.05 using ANOVA post-hoc Tukey’s test.

Figure 1 showed changes in water content (control vs. SA treated) samples in different three seasons during rehydration period. Water content was expressed as a percentage of that at full turgor. In all the seasons, the water content (%) in the SA treatment was found to be higher as compared to their respective controls at 0 h. In the case of SA treated, specifically, the trend of the water content increased at 12 h and steadily decreased as the time progressed, while in the case of control samples the trend was variable.

### 2.2. Effect of SA and Seasonal Variation on Chlorophyll a Fluorescence Parameters

**Figure 2:**
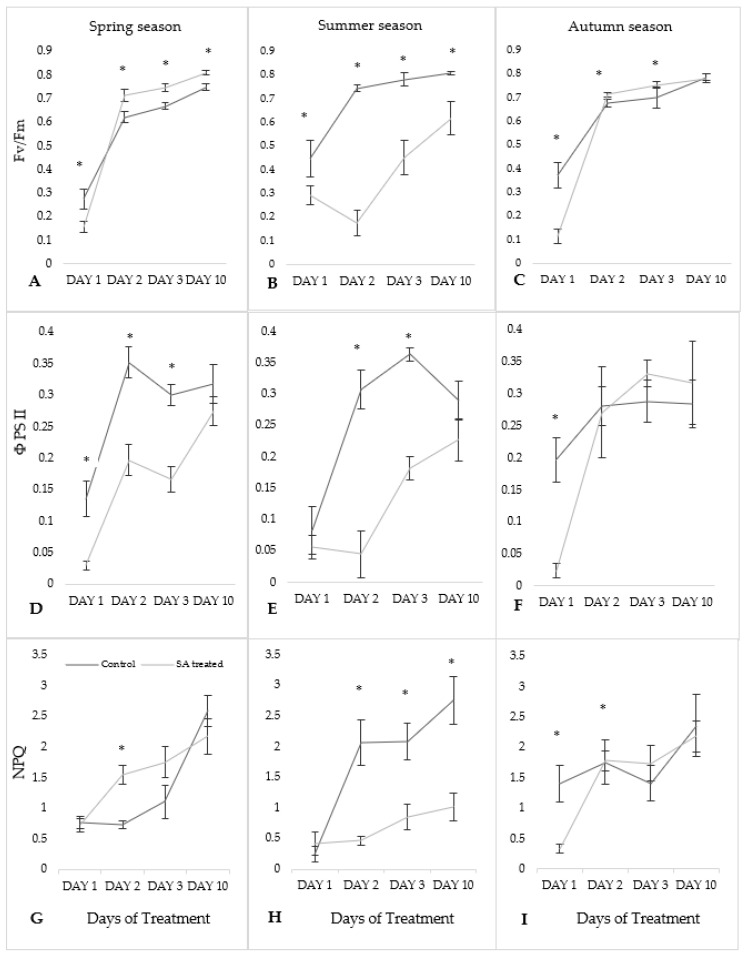
Changes of kinetic of chlorophyll a fluorescence (Fv/Fm: (**A**–**C**); ΦPSII: (**D**–**F**); NPQ: (**G**–**I**)) in *S. ruralis* after SA treatment respect to different season (Spring, Summer, Autumn). The mean values (*n* = 3) ± SD marked with an asterisk (*) are significantly different at *p* ≤ 0.05 using ANOVA post-hoc Tukey’s test.

Chlorophyll *a* fluorescence parameter values of SA-treated moss cushions were compared with control samples in different seasons. Fv/Fm values were found to be significantly different (*p* ≤ 0.05) compared to the mean values with Days of treatment in each season except for the autumn season on Day 10 (Figure 2A–C). In the spring season, ΦPSII values were shown significantly in all three Days except on Day 10 whereas in summer season Day 1 and Day 10 were found not significant. In the autumn season, Day 1 showed significant differences, but other Days were not significantly different (Figure 2D–F). NPQ values were significantly different on Day 2 in spring season and Day 2, 3, 10, in the summer season whereas in autumn season on Day 1, 2 (Figure 2G–I).

Fv/Fm parameter values of SA-treated samples were found lower on Day 1 in each season as compared to the control values with other Days of treatment (Figure 2A–C). Spring and autumn season were slightly varied to each other but in the summer season, higher variation was found. In the spring season, the lowest Fv/Fm values in SA-treated samples were found on Day 1 (0.157 ± 0.02) and the highest on Day 10 (0.810 ± 0.01). On Day 1, SA-treated samples for spring and autumn season were lower than the summer season. However, from Day 2 to Day 10 observed values were lower in the summer season as compared with the control values. In the summer season, lowest values were observed on Day 2 (0.173 ± 0.05) and highest on Day 10 (0.614 ± 0.07). In the autumn season, the lowest was on Day 1 (0.112 ± 0.03) and highest on Day 10 (0.780 ± 0.01).

For ΦPSII parameter, SA-treated values were found lower on Day 1 and then increased until Day 10 in the spring season (Figure 2D–F). The lowest value was recorded on Day 1 (0.030 ± 0.007) and highest on Day 10 (0.274 ± 0.05). Initially, values recorded in the summer season was decreased till Day 2 and then showed inclined pattern. SA-treated samples were found lowest on Day 2 (0.045 ± 0.037) and highest on Day 10 (0.227 ± 0.07). In the autumn season, the lowest values were observed on Day 1 (0.024 ± 0.01) while the highest on Day 3 (0.332 ± 0.02) for SA-treated samples. From Day 1, ΦPSII values increased continuously till Day 10.

NPQ values were slightly varied to each other during spring and autumn season whereas higher variation was seen in the summer season (Figure 2G–I). Lowest NPQ values were found on Day 1 for SA-treated samples in each season (0.726 ± 0.11) for the spring season, for the summer season (0.415 ± 0.19) and autumn season (0.337 ± 0.07). Highest values for SA-treated samples were recorded on Day 10 in each season (2.171 ± 0.28) for the spring season, (1.019 ± 0.22) for the summer season and autumn season (2.185 ± 0.25).

### 2.3. Protein Determination

**Figure 3:**
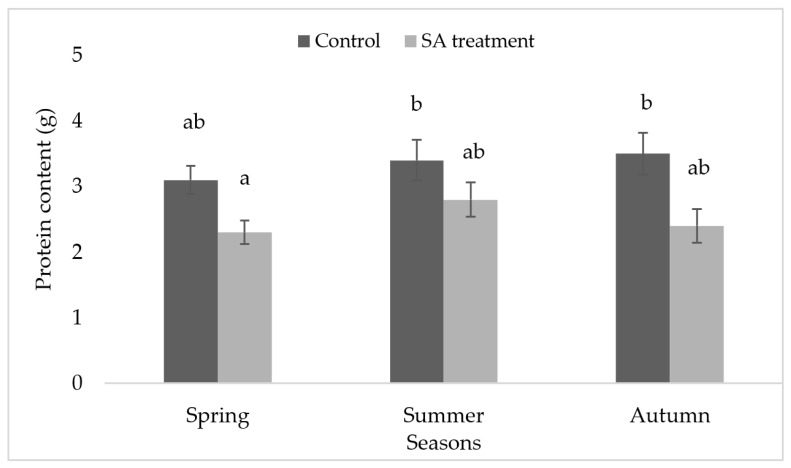
Protein content in *S. ruralis* with respect to different seasons (Spring, Summer, Autumn). The mean values (*n* = 5) ± SD with different alphabetical letters are significantly different at *p* ≤ 0.05 using ANOVA post-hoc (Tukey’s test).

Mean values were calculated and ANOVA post-hoc Tukey’s test was performed to find out the difference in the mean protein content between the control and SA treatment in Figure 3. There was no significant difference either season-wise or in SA treatment.

### 2.4. Effect of SA Treatment on Antioxidant Enzymatic Activity

**Figure 4:**
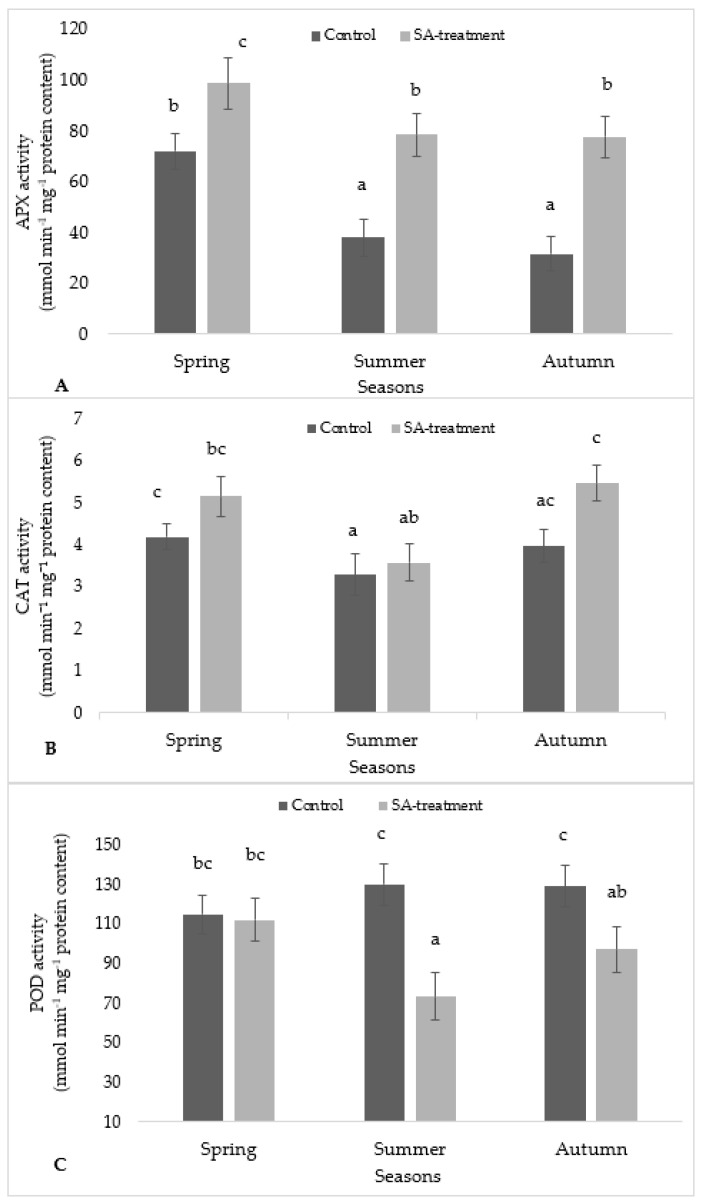
The effect of SA treatment on the activity of antioxidant enzymes in *S. ruralis*: (**A**) APX; (**B**) CAT; (**C**) guaiacol peroxidase (POD) with respect to different seasons (Spring, Summer, Autumn). Mean values (*n* = 5) ± SD marked with different alphabetical letter are significantly different at *p* ≤ 0.05 using ANOVA post-hoc (Tukey’s test).

Antioxidant enzymatic activity results were represented in two different ways, i.e., treatment-wise (control and SA treated) within the same season and season-wise in Figure 4. Based on treatment wise for the APX activity (Figure 4A), there was a significant difference between the control and SA-treated for spring, summer, and autumn seasons whereas on the basis of seasons, the APX activity in the case of control and SA in spring was significantly different to summer and autumn seasons, respectively. No significant difference was observed between summer and autumn.

The CAT activity in SA-treated samples was very slightly increased as compared to their respective control values in spring, autumn, and summer (Figure 4B). A season-wise comparison showed CAT activity in control is significantly different to CAT activity in summer, while the CAT activity in SA-treated in autumn was significantly different to that in summer.

The POD activity in SA-treated in spring was significantly different to that in SA-treated in summer (Figure 4C). Based on treatment-wise, no significant difference was observed between control and SA-treated in spring season, respectively except summer and autumn season.

## 3. Discussion

In this study, we examined how the effects of SA pre-treatment relates to photosynthetic efficiency using chlorophyll fluorometer and determined the antioxidant enzymatic activities. Seasonal variation was observed and studied in moss cushions collected from three different seasons. Moss cushions were monitored for three consecutive Days and till Day 10 under control and SA-treated conditions. The ratio of variable and maximum fluorescence in a dark-adapted state (Fv/Fm) is used as an important and sensitive indicator of plant photosynthetic performance in chlorophyll fluorescence measurement. It indicates the maximum efficiency of PS II when all PS II centers are open. Fv/Fm values are found in the range of 0.79 to 0.83 approximate optimal values in most plant species and lowered values indicating the condition of plant stress [14]. The results showed an inclined trend from Day 1 till Day 10 in SA-treated samples while comparing with control samples in spring and autumn season in Figure 2A,C. These increased fluctuations indicated the recovery of moss cushions after a long term of desiccation within each season in a different way. However, in summer season, declined pattern was observed in the Fv/Fm values in Figure 2B for SA-treated samples. In spring and summer season, ΦPSII values showed a declined pattern for SA-treated samples as compared to control in Figure 2D,E. However, opposite trend was observed in autumn season in Figure 2F. It might indicate some protective mechanism towards photo inhibitory damage in response to stress could be due to combined effect of SA treatment and high temperature during (July 2018) period of collection which might reduce the photosynthetic efficiency. In general, fewer reaction centers were opened during that time which lowered the Fv/Fm ratio and the plant may be experienced greater stress [15].

For non-photochemical quenching parameters, SA-treated samples showed the higher values of NPQ during the spring and autumn season but lower in the summer season. Similar results were reported in [16] where NPQ values were shown higher ABA hormone pre-treatment in the moss *Atrichum undulatum* (Hedw.) P. Beauv. and *A. androgyne* (C. Müll.) A. Jaeger [17]. NPQ reflects the level of energy dissipation in the form of heat energy. During rehydration, its increase provides benefit to the moss cushions by the heat dissipation in excess or photoprotection indicates the higher level of desiccation tolerance in *S. ruralis* moss cushions. But it is still not clear how NPQ is related to SA tolerance levels.

Protein content was observed decreased in SA-treated as compared to control (Figure 3). It may indicate the protein denaturation due to the inhibitory response of SA in different seasonal conditions. In the desiccated state, accumulation of ROS increases the damage to proteins and lipids in the chloroplast also in mitochondria, peroxisomes, and plasma membrane. ROS causes inhibition of protein synthesis or protein denaturation [18]. However, there is a down-regulation of the synthesis of proteins during drying conditions [19].

Catalase activity showed slightly variation in all seasons but significantly not different between SA-treatment and control. Ascorbate peroxidase under SA-treatment were found to be increased which caused a decrease in oxidative stress during each season (Figure 4). This might be related to the protective mechanism against the stressful conditions caused due to pre-treatment of SA compared with control values. Another unfavorable conditions occurred may be due to temperature and water stress depending on different seasons. According to [20], low temperature and water stress leads to overproduction of ROS that causes the oxidative damage to the cells. Moss cushions might be induced these enzymes to scavenge ROS and enhancing their tolerance during the different seasons [21]. Hydrogen peroxide is produced during oxidative stress caused by the overproduction of ROS is decomposed by peroxidase enzymes [22]. An earlier study has been reported that several bryophytes showed significant antioxidant activity and possessed with efficient antioxidant enzyme systems. Antioxidant peroxidase was characterized in the liverwort *Marchantia polymorpha* L. that found different from other known peroxidases in vascular plants [23]. Similarly, the role of ascorbate peroxidase was found in the removal of hydrogen peroxide in a moss *Brachythecium velutinum* (Hedw.) Schimp. and *M. polymorpha* [24]. Guaiacol peroxidase was found to be higher in control moss cushions as compared to SA-treated (Figure 4). In earlier study, higher guaiacol peroxidase activity was reported in the two liverwort species *Plagiochasma appendiculatum* Lehm. and Lindenb. and *Pellia endivaefolia* (Dicks.) Dumort. indicating the role of these enzymes and participation in the antioxidant system [25].

Our results also showed that SA pre-treatment has a significant effect on photosystem II in the moss *S.ruralis*. This would be the first study of SA pre-treatment on *S. ruralis* to explore the mechanism behind the defense system in bryophytes. It contributes to a better understanding of abiotic interaction in non-vascular plants. Increased activity of antioxidant enzymes has been suggested as an adaptive protective mechanism against oxidative damage due to pre-treatment of SA. It can be concluded that seasonal variation has been observed in chlorophyll fluorescence and antioxidant system after long term of desiccation in *S. ruralis* species that could be because of SA and might be due to fluctuations in conditions of their habitat, duration of light intensity, temperature and precipitation. The present work can be useful in finding the role of the SA hormone in bryophytes.

## 4. Materials and Methods

### 4.1. Collection of Plant Material

The moss cushions of *S. ruralis* were collected from flat areas of semi-arid sandy grassland near Bócsa-Bugac in Kiskunság region (central Hungary 46°53′29″ N, 19°26′35.6″ E) in an air-dried state. These sandy grasslands are part of the Kiskunság National Park, Bugac in the Hungarian Great Plain. Samples were collected in three different seasons: Spring (May 2018), summer (July 2018), and autumn (October 2018). Climatic conditions of these grasslands were recorded, and monthly average meteorological parameters were mentioned in [11].

### 4.2. Experimental Set-Up

In the preliminary study, we examined chlorophyll fluorescence under different SA concentrations: 10 µM (low), 0.001 M, (medium), 0.01 M (high). Medium concentration was selected to conduct the final experiment. Moss cushions of *S. ruralis* were cleaned and transferred to large size Petri dishes nearby to the window after one year of collection period. Samples were divided into 6 Petri-dishes included 3 for control (distilled water treatment) and 3 for SA treatment. Samples were rehydrated by placing them in Petri dishes under SA treatment for 72 h. Water content (WC%) was calculated by WC = [(FW−DW)/DW] ×100, where FW is the fresh weight while DW is the oven-dried weight of the sample.

### 4.3. Measurements of Chlorophyll a Fluorescence Parameter

At the beginning of the experiment, samples were sprayed with distilled water for control Petri-dishes and SA solution (0.001 M) for SA Petri-dishes. After 6 h of rehydration, chlorophyll fluorescence was measured daily on all the samples from different seasons until 3 Days and then on the 10th Day. Hansatech pulse modulated chlorophyll fluorometer (FMS 2) was used to measure the chlorophyll fluorescence and to describe the functioning of photosynthetic apparatus three chlorophyll fluorescence parameters were chosen: Maximum photochemical quantum yield of PS II (Fv/Fm), effective photochemical quantum yield of PS II (ΦPSII), non-photochemical quenching (NPQ).

### 4.4. Enzyme Extraction and Spectrophotometric Antioxidant Enzymatic Assays

To determine the activity of antioxidant enzymes, 0.3 g moss shoots (control and SA-treated) were ground to a fine powder in liquid nitrogen and homogenized in 2 mL of potassium phosphate extraction buffer (125 mM, pH = 7.8) using a pre-chilled mortar and pestle. The extract was centrifuged at 4 °C for 10 min at 15,000 rpm in a cooling centrifuge (HERMLE Z216 MK). The supernatant was used to determine the activity of ascorbate peroxidase (APX; EC 1.11.1.11), catalase (CAT; EC 1.11.1.6), and guaiacol peroxidase (POD; EC 1.11.1.7) according to [26] with some modifications. Molar extinction coefficient (ε) was used to calculate the enzymatic activities and expressed in terms of mmol min^−1^ mg^−1^ protein content. Antioxidant enzymatic activities were evaluated, and protein content was calculated after 72 h of treatment.

APX reaction mixture consisted of 125 mM potassium phosphate buffer (pH = 7.0), 5 mM Na-ascorbate, 1 mM Na_2_-EDTA, 100 mM H_2_O_2_ and 0.1 mL plant enzyme extract was completed to a final volume 1mL at 25 °C. Enzyme activity was assayed by following the decrease in absorbance at 290 nm for 100 s (ε = 2.8 mM^−1^ cm^−1^).

CAT activity was determined by measuring the decrease in the H_2_O_2_ concentration at absorbance 240 nm for 340 s (ε = 36.6 mM^−1^ cm^−1^). The enzyme activity was assayed in a 1 mL reaction mixture consisting of 125 mM potassium phosphate buffer (pH = 7.0), 100 mM H_2_O_2_ and 0.1 mL plant enzyme extract were added to initiate the reaction.

POD activity was assayed in 1 mL reaction mixture consists of 125 mM potassium phosphate buffer (pH = 7.0), 34 mM guaiacol, 100 mM H_2_O_2_, 0.1 mL plant enzyme extract at 25 °C. Enzyme activity was determined by the increase in absorbance at 470 nm for 150 s (ε = 36.6 mM^−1^ cm^−1^). Tetra guaiacol concentration was increased in a reaction mixture because of guaiacol oxidation.

### 4.5. Protein Determination

Protein determination was performed to calculate the enzymatic activity based on protein content. The concentration of protein was determined according to [27] with some modifications. Bovine serum albumin (BSA) was used to prepare the standard curve. Protein content was measured based on the reaction of the Coomassie Blue G-250 dye with proteins with extinction coefficient at 595 nm (ε = 43,000 M^−1^ cm^−1^). Enzyme extracts from control and SA-treated were measured spectrophotometrically (SHIMADZU UV-1061 UV-visible spectrophotometer) at 595 nm wavelength.

### 4.6. Statistical Analysis

Statistical analyses were performed using the statistical software R programming language version 3.5.3 for Windows (R development Core Team, Auckland, New Zealand). ANOVA parametric test was used to check the significant differences with different seasons (spring, summer, Autumn). ANOVA post-hoc (Tukey’s test) was performed at 95% confidence level to determine the significant differences between each pair of seasons with different chlorophyll *a* parameters and antioxidant enzymatic activities. Differences at *p* ≤ 0.05 were considered statistically significant.

## Abbreviations

ANOVA	one-way analysis of variance
APX	ascorbate peroxidase
CAT	catalase
Chl	chlorophyll
Fv/Fm	maximum photochemical quantum yield of PS II
NPQ	non-photochemical quenching
PS II	photosystem II
ΦPSII	Effective photochemical quantum yield of PSII
POD	guaiacol peroxidase
ROS	reactive oxygen species
SA	salicylic acid
WC	water content
Ε	molar extinction coefficient
